# Comparing serotype coverage of pneumococcal vaccines with PCV21 (V116), a new 21-valent conjugate pneumococcal vaccine, and the epidemiology of its eight unique *Streptococcus pneumoniae* serotypes (15A, 15C, 16F, 23A, 23B, 24F, 31 and 35B) causing invasive pneumococcal disease in adult patients in Canada: SAVE study, 2018–21

**DOI:** 10.1093/jac/dkaf085

**Published:** 2025-03-25

**Authors:** John J Schellenberg, Heather J Adam, Melanie R Baxter, James A Karlowsky, Alyssa R Golden, Irene Martin, George G Zhanel

**Affiliations:** Department of Medical Microbiology and Infectious Diseases, Max Rady College of Medicine, Room 543-745 Bannatyne Avenue, Winnipeg, Manitoba R3E 0J9, Canada; Department of Medical Microbiology and Infectious Diseases, Max Rady College of Medicine, Room 543-745 Bannatyne Avenue, Winnipeg, Manitoba R3E 0J9, Canada; Clinical Microbiology, Shared Health, MS673-820 Sherbrook Street, Winnipeg, Manitoba R3A 1R9, Canada; Department of Medical Microbiology and Infectious Diseases, Max Rady College of Medicine, Room 543-745 Bannatyne Avenue, Winnipeg, Manitoba R3E 0J9, Canada; Department of Medical Microbiology and Infectious Diseases, Max Rady College of Medicine, Room 543-745 Bannatyne Avenue, Winnipeg, Manitoba R3E 0J9, Canada; Clinical Microbiology, Shared Health, MS673-820 Sherbrook Street, Winnipeg, Manitoba R3A 1R9, Canada; National Microbiology Laboratory, Public Health Agency of Canada, 1015 Arlington Street, Winnipeg, Manitoba R3E 3R2, Canada; National Microbiology Laboratory, Public Health Agency of Canada, 1015 Arlington Street, Winnipeg, Manitoba R3E 3R2, Canada; Department of Medical Microbiology and Infectious Diseases, Max Rady College of Medicine, Room 543-745 Bannatyne Avenue, Winnipeg, Manitoba R3E 0J9, Canada

## Abstract

**Background:**

V116 is a novel 21-valent pneumococcal conjugate vaccine (PCV) intended for use in adults.

**Objectives:**

To estimate current V116 serotype coverage in adult patients in Canada compared with PCV15, PCV20 and PPSV23 vaccines, and to describe isolate demographics for the eight unique serotypes (15A, 15C, 16F, 23A, 23B, 24F, 31 and 35B) covered by V116.

**Methods:**

From 2018 to 2021 inclusive, the SAVE study collected 5854 invasive pneumococcal disease (IPD) isolates as part of a collaboration between the Canadian Antimicrobial Resistance Alliance and the Public Health Agency of Canada–National Microbiology Laboratory. Serotypes were determined by Quellung reaction and antimicrobial susceptibility testing performed using the CLSI broth microdilution method.

**Results:**

For adult patients (≥18 years), adults 50–64 years and adults ≥65 years, respectively, IPD isolate coverage was PCV15 (42.7%; 41.0%, 39.8%), PCV20 (59.0%; 60.2%, 52.2%), PPSV23 (70.4%; 75.1%, 60.0%), V116 (78.9%; 76.3%, 81.5%) and V116 plus PCV20 (92.2%; 91.0%, 89.3%). The eight unique V116 serotypes accounted for 19.7% and 26.8% of IPD isolates from adults and adults ≥65 years, respectively. Among the eight unique V116 serotypes, 15A and 23A demonstrated the highest rates of MDR (17.0% and 10.2%, respectively); 6.7% of 15A isolates were XDR.

**Conclusions:**

V116 provided significantly (*P *< 0.05) greater coverage than PCV15, PCV20 and PPSV23 for adults, including older adults, across all Canadian geographic regions, and against IPD isolates with common antimicrobial resistance phenotypes, including MDR. The eight unique V116 serotypes accounted for a higher proportion of IPD isolate serotypes in patients aged ≥65 years than younger adults.

## Introduction

Pneumococcal conjugate vaccines (PCVs) have demonstrated effectiveness in preventing pneumococcal pneumonia and invasive pneumococcal disease (IPD), especially in patients at highest risk (i.e. children <2 years of age and adults ≥65 years of age), and have reduced the usage of antimicrobial agents.^1–7^ It is important to monitor the evolution of pneumococcal vaccine serotypes as vaccine formulations with expanded coverage become available in North America, Europe and across the world. Appreciating the current distribution of IPD serotypes aids in vaccine coverage prediction for newly introduced formulations.

In 2000, the first PCV (PCV7, Prevnar^®^) was introduced. It provided stronger and more long-lasting protection than polysaccharide vaccines via stimulation of prolonged memory T cells. PCV7 covered 7 of the 23 serotypes contained in pneumococcal polysaccharide vaccine 23 (PPSV23).^6,7^ PCV7 was followed by newer conjugated vaccines with increasing valency: PCV10, PCV13, PCV15 and most recently PCV20.^8–11^

PCV15 (Vaxneuvance^®^) and PCV20 (Prevnar^®^-20) were approved for use in Canada, the USA, and several other countries worldwide based largely on immunogenicity data.^12,13^ In 2024, the US Advisory Committee on Immunization Practices (ACIP) recommended the use of PCV15 (followed by PPSV23) or PCV20 in PCV-naive adults aged ≥65 years or 18–64 years with certain underlying conditions.^13^ In 2023, Canada’s National Advisory Committee on Immunization (NACI) made the preferential recommendation of a single dose of PCV20, and indicated that PCV15 (followed by PPSV23) should be reserved for use in individuals in three patient groups (≥65 years, 50–64 years with risk factors, and ≥18 years with immunocompromising conditions) only when PCV20 is not available.^12^ Currently, due to its only recent introduction to the Canadian market, its only recent NACI recommendation to be used in adults, and until recently its lack of government reimbursement for the vaccine, the usage of PCV20 in Canadian adults currently represents only a small fraction of the various pneumococcal vaccines used in adults.

V116 is a novel 21-valent PCV (PCV21) developed specifically for use in adults, covering serotypes first included in PCV13 (3, 6A, 7F, 19A), PCV15 (22F, 33F), PCV20 (8, 10A, 11A, 12F) and PPSV23 (9N, 17F, 20), as well as eight unique serotypes (15A, 15C, 16F, 23A, 23B, 24F, 31, 35B) not included in any currently licensed pneumococcal vaccine.^14–21^ The serotypes in V116 were selected for inclusion based, in part, on the evaluation of available global epidemiology data in older adults following introduction of PCVs into both paediatric and adult national immunization plans.^14,18,21^ The eight unique serotypes in V116 substantially contribute to current adult pneumococcal disease burden. V116, also known as PCV21, was approved by the US FDA in June 2024. Subsequently, ACIP recommended PCV21 as an option for adults aged ≥19 years who are currently recommended to receive a dose of a PCV.^22^ It should be mentioned that PCV20 contains nine serotypes (excluding 15B/C)—1, 4, 5, 6B, 9V, 14, 18C, 19F and 23F—that are not contained in PCV21 (V116).

The purpose of the current study was to assess V116 (PCV21) serotype coverage in adult patients with IPD in Canada from 2018 to 2021 inclusive, compared with the coverage offered by earlier PCV15, PCV20 and PPSV23 vaccines, and to describe isolate demographics for the eight unique serotypes covered by V116.

## Materials and methods

### Bacterial isolates

The IPD isolates tested in this study were from the *Streptococcus pneumoniae* Serotyping and Antimicrobial Susceptibility: Assessment for Vaccine Efficacy in Canada (SAVE) study, an annual national study from 2011 to 2021 inclusive, focused on characterizing invasive isolates of *S. pneumoniae* obtained across Canada after the introduction of PCV13.^7,8,23–26^ The SAVE study is a collaboration between the Canadian Antimicrobial Resistance Alliance (CARA) and the Public Health Agency of Canada–National Microbiology Laboratory (PHAC-NML).^23–27^ The SAVE study received 5854 IPD isolates from 2018 to 2021 inclusive. *S. pneumoniae* isolates from sterile sites were initially forwarded from Canadian public health laboratories [Canadian Public Health Laboratory Network (CPHLN)] to PHAC–NML. After permission was received from submitting CPHLN sites by PHAC–NML (as detailed in the Acknowledgments section), the *S. pneumoniae* isolates were forwarded to CARA for testing, as previously described.^7,8,23–26^

### Serotyping

Serotyping was performed using the Quellung reaction with pool/group/type/factor commercial antisera (SSI Diagnostica; Statens Serum Institut, Copenhagen, Denmark), as previously described.^7,8,27^ Serotypes 15B and 15C were grouped together due to the reported reversible switching between these serotypes and documented cross-reactive functional antibodies, and serotypes 6A and 6C were grouped together due to cross-reactive functional antibodies.^15,28,29^ It should be mentioned at this time that no data exist on whether the immunological cross-reactive antibodies of serotypes 15B and 15C result in clinical cross-protection.

### Antimicrobial susceptibility testing

Antimicrobial susceptibility testing was performed using custom-designed, broth microdilution panels produced in house using CLSI standard methods, including quality control, as previously described.^8,23,24^ Isolates were classified as susceptible, intermediate or resistant according to CLSI breakpoints for seven antimicrobial agents: chloramphenicol, clindamycin, clarithromycin, doxycycline, levofloxacin, penicillin and trimethoprim/sulfamethoxazole. Isolates defined as not susceptible were a combination of intermediate and resistant phenotypes. MDR was defined by resistance to three or more antimicrobial classes and XDR defined by resistance to five or more antimicrobial classes. It should be stated that other definitions of MDR and XDR *S. pneumoniae* exist.^23^ Penicillin resistance was defined as an MIC of ≥2 mg/L.^8,23,24^

### Statistical analysis

Figures were generated using custom Python and R scripts. Statistically significant differences (*P *< 0.05) in vaccine coverage of isolates by demographic category and coverage of MDR by vaccine V116 versus PCV20, V116 versus PPSV23, and PCV20 versus PPSV23 were determined using the two-tailed Fisher’s exact test (α = 0.05), using custom scripts in R.

## Results

### IPD isolate coverage by V116 and comparator vaccines

Table 1 shows the proportion of IPD isolates with V116 serotypes (vaccine coverage) compared with proportions for PCV13, PCV15, PCV20, PPSV23, and V116 combined with PCV20, stratified by collection year, patient age group, Canadian geographic region and biological sex. It should be mentioned that data on the collection year, patient age group, Canadian geographic region and biological sex were not available for all isolates. V116 provided significantly greater coverage of IPD isolates than PCV20 and PPSV23 for most age groups (including patients aged 50–64 and ≥65 years), across all Canadian geographic regions, and for both biological sexes. V116 also compared favourably regarding coverage of IPD isolates versus PCV15/PPSV23 in patients aged 50–64 (76.3% versus 76.5%, respectively) and ≥65 years (81.5% versus 62.1%, respectively).

**Table 1.** Proportion of invasive *S. pneumoniae* isolates with V116 serotypes compared with other vaccine formulations (PCV13, PCV15, PCV20, PPSV23) and V116 combined with PCV20 by collection year, patient age, Canadian geographic region and biological sex

Category (*n*)	Proportion of isolates with vaccine serotype, *n* (%)						*P* value		
	PCV13	PCV15	PCV20	PPSV23	V116	PCV20 and V116	V116 versus PCV20	V116 versus PPSV23	PCV20 versus PPSV23
All isolates (5854)	1719 (29.4)	2387 (40.8)	3443 (58.8)	4121 (70.4)	4442 (75.9)	5282 (90.2)	<0.0001	<0.0001	<0.0001
Year									
2018 (607)	140 (23.1)	234 (38.6)	330 (54.4)	352 (58.0)	452 (74.5)	526 (86.7)	<0.0001	<0.0001	NS^a^
2019 (1332)	506 (38.0)	613 (46.0)	922 (69.2)	1103 (82.8)	963 (72.3)	1260 (94.6)	NS	<0.0001	<0.0001
2020 (1696)	533 (31.4)	695 (41.0)	1021 (60.2)	1273 (75.1)	1294 (76.3)	1543 (91.0)	<0.0001	NS	<0.0001
2021 (2161)	521 (24.1)	821 (38.0)	1133 (52.4)	1350 (62.5)	1689 (78.2)	1901 (88.0)	<0.0001	<0.0001	<0.0001
Age group, years									
0 to <18 (607)	140 (23.1)	234 (38.6)	330 (54.4)	352 (58.0)	452 (74.5)	526 (86.7)	<0.0001	<0.0001	NS
18–49 (1332)	506 (38.0)	613 (46.0)	922 (69.2)	1103 (82.8)	963 (72.3)	1260 (94.6)	NS	<0.0001	<0.0001
50–64 (1696)	533 (31.4)	695 (41.0)	1021 (60.2)	1273 (75.1)	1294 (76.3)	1543 (91.0)	<0.0001	NS	<0.0001
≥65 (2161)	562 (26.0)	860 (39.8)	1128 (52.2)	1297 (60.0)	1761 (81.5)	1930 (89.3)	<0.0001	<0.0001	<0.0001
Region^b^									
Western (1307)	403 (30.8)	543 (41.5)	842 (64.4)	1020 (78.0)	956 (73.1)	1225 (93.7)	<0.0001	<0.005	<0.0001
Central (4128)	1225 (29.7)	1690 (40.9)	2373 (57.5)	2801 (67.9)	3149 (76.3)	3682 (89.2)	<0.0001	<0.0001	<0.0001
Eastern (419)	91 (21.7)	154 (36.8)	228 (54.4)	300 (71.6)	337 (80.4)	375 (89.5)	<0.0001	<0.005	<0.0001
Biological sex									
Female (2415)	683 (28.3)	989 (41.0)	1415 (58.6)	1666 (69.0)	1858 (76.9)	2174 (90.0)	<0.0001	<0.0001	<0.0001
Male (3140)	926 (29.5)	1247 (39.7)	1825 (58.1)	2220 (70.7)	2354 (75.0)	2828 (90.1)	<0.0001	<0.0001	<0.0001

^a^NS, not significant.

^b^Western Canada includes Saskatchewan and Manitoba; Central Canada includes Ontario and Quebec; Eastern Canada includes Newfoundland and Labrador, Nova Scotia, Prince Edward Island and New Brunswick.

**Figure 1.**
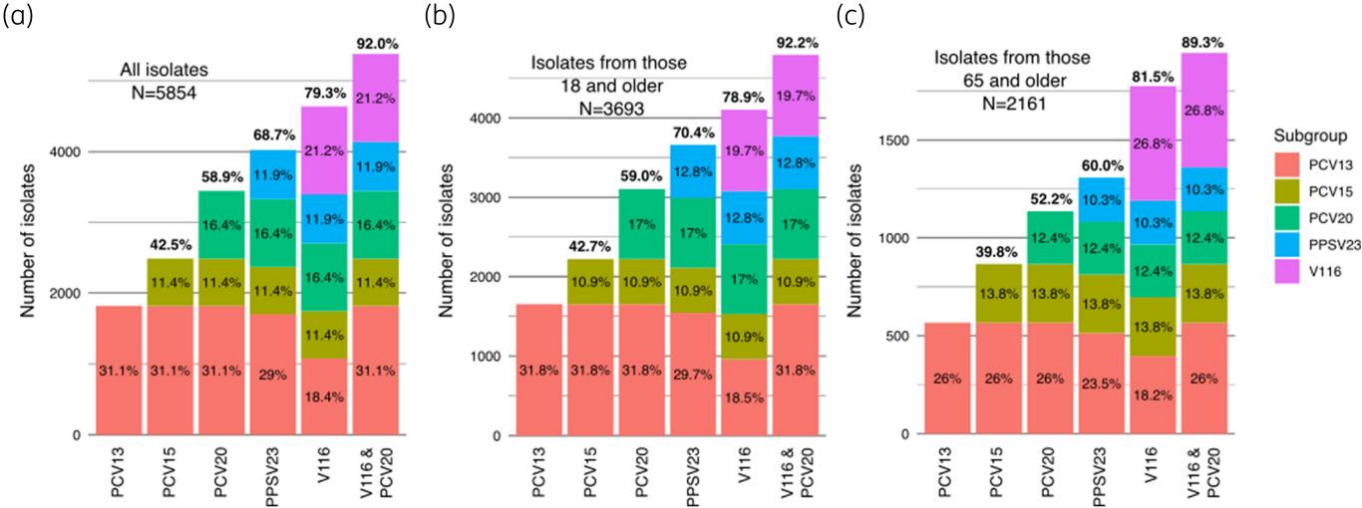
IPD isolate coverage by vaccine formulation (PCV13, PCV15, PCV20, PPSV23, V116 and V116 combined with PCV20) and proportion of isolates covered by serotypes first introduced or unique to each vaccine formulation (subgroup) for (a) all isolates, (b) isolates from patients aged ≥18 years, and (c) isolates from patients aged ≥65 years.

Vaccine coverage of PCV13, PCV15, PCV20, PPSV23, V116 alone, and V116 combined with PCV20 for all isolates, for isolates from patients aged ≥18 years, and for isolates from patients aged ≥65 years is also depicted in Figure 1a–c. For adult patients, vaccine coverage was 42.7% for PCV15, 59.0% for PCV20, 70.4% for PPSV23, 78.9% for V116 and 92.2% for PCV20 combined with V116. In patients aged ≥65 years, vaccine coverage was 39.8% for PCV15, 52.2% for PCV20, 60.0% for PPSV23, 81.5% for V116 and 89.3% for V116 combined with PCV20. The non-PCV21 (non-V116) serotypes contained in PCV20 accounted for 13.7% of all serotypes in ages 50–64 years and 7.6% of all serotypes in ≥65 years.

### Vaccine coverage of antimicrobial-resistant and MDR isolates by V116 and comparator vaccines

Table 2 summarizes the proportions of IPD isolates with V116 serotypes (vaccine coverage) compared with PCV13, PCV15, PCV20, PPSV23, and V116 combined with PCV20 by antimicrobial resistance phenotype. V116 provided significantly greater coverage than both PCV20 and PPSV23 for isolates resistant to clindamycin, clarithromycin, doxycycline or penicillin, as well as against MDR and XDR isolates. V116 provided coverage of 56.3%–84.4% of serotypes with resistance to one antimicrobial (chloramphenicol, clindamycin, clarithromycin, doxycycline, levofloxacin, penicillin or trimethoprim/sulfamethoxazole); 72.9% coverage of MDR isolates and 72.0% coverage of XDR isolates. Figure S1(a and b) (available as Supplementary data at *JAC* Online) provides greater detail regarding serotypes unique to V116 and PCV20, and serotypes shared by V116 and PPSV23, and V116 and PCV20 including antimicrobial-resistant phenotypes.

**Table 2.** Proportion of invasive *S. pneumoniae* isolates with V116 serotypes, compared with other vaccine formulations (PCV13, PCV15, PCV20, PPSV23) and V116 combined with PCV20, stratified by antimicrobial resistance phenotypes

Category (*n*)	Proportion of isolates with vaccine serotype, *n* (%)						*P* value		
	PCV13	PCV15	PCV20	PPSV23	V116	PCV20 and V116	V116 versus PCV20	V116 versus PPSV23	PCV20 versus PPSV23
CHL-R (233)	125 (53.6)	144 (61.8)	188 (80.7)	191 (82.0)	179 (76.8)	223 (95.7)	NS^a^	NS	NS
CLI-R (410)	177 (43.2)	201 (49.0)	216 (52.7)	229 (55.9)	313 (76.3)	386 (94.1)	<0.0001	<0.0001	NS
CLR-R (1381)	326 (23.6)	727 (52.6)	926 (67.1)	1008 (73.0)	1165 (84.4)	1302 (94.3)	<0.0001	<0.0001	<0.001
DOX-R (600)	248 (41.3)	264 (44.0)	357 (59.5)	381 (63.5)	457 (76.2)	556 (92.7)	<0.0001	<0.0001	NS
LVX-R (16)	4 (25.0)	5 (31.3)	6 (37.5)	8 (50.0)	9 (56.3)	12 (75.0)	NS	NS	NS
PEN-R (170)	87 (51.2)	89 (52.4)	94 (55.3)	95 (55.9)	121 (71.2)	160 (94.1)	<0.005	<0.005	NS
SXT-R (422)	108 (25.6)	150 (35.5)	257 (60.9)	267 (63.3)	265 (62.8)	319 (75.6)	NS	NS	NS
Any resistance (1736)	411 (23.7)	822 (47.4)	1170 (67.4)	1221 (70.3)	1391 (80.1)	1557 (89.7)	<0.0001	<0.0001	NS
MDR (442)	186 (42.1)	204 (46.2)	242 (54.8)	256 (57.9)	322 (72.9)	413 (93.4)	<0.0001	<0.0001	NS
XDR (75)	60 (80.0)	62 (82.7)	69 (92.0)	68 (90.7)	54 (72.0)	74 (98.7)	<0.005	<0.005	NS

CHL-R, chloramphenicol resistant; CLI-R, clindamycin resistant; CLR-R, clarithromycin resistant; DOX-R, doxycycline resistant; LVX-R, levofloxacin resistant; PEN-R, penicillin resistant; SXT-R, trimethoprim/sulfamethoxazole resistant.

^a^NS, not -significant.

### The eight unique serotypes covered by V116

**Figure 2.**
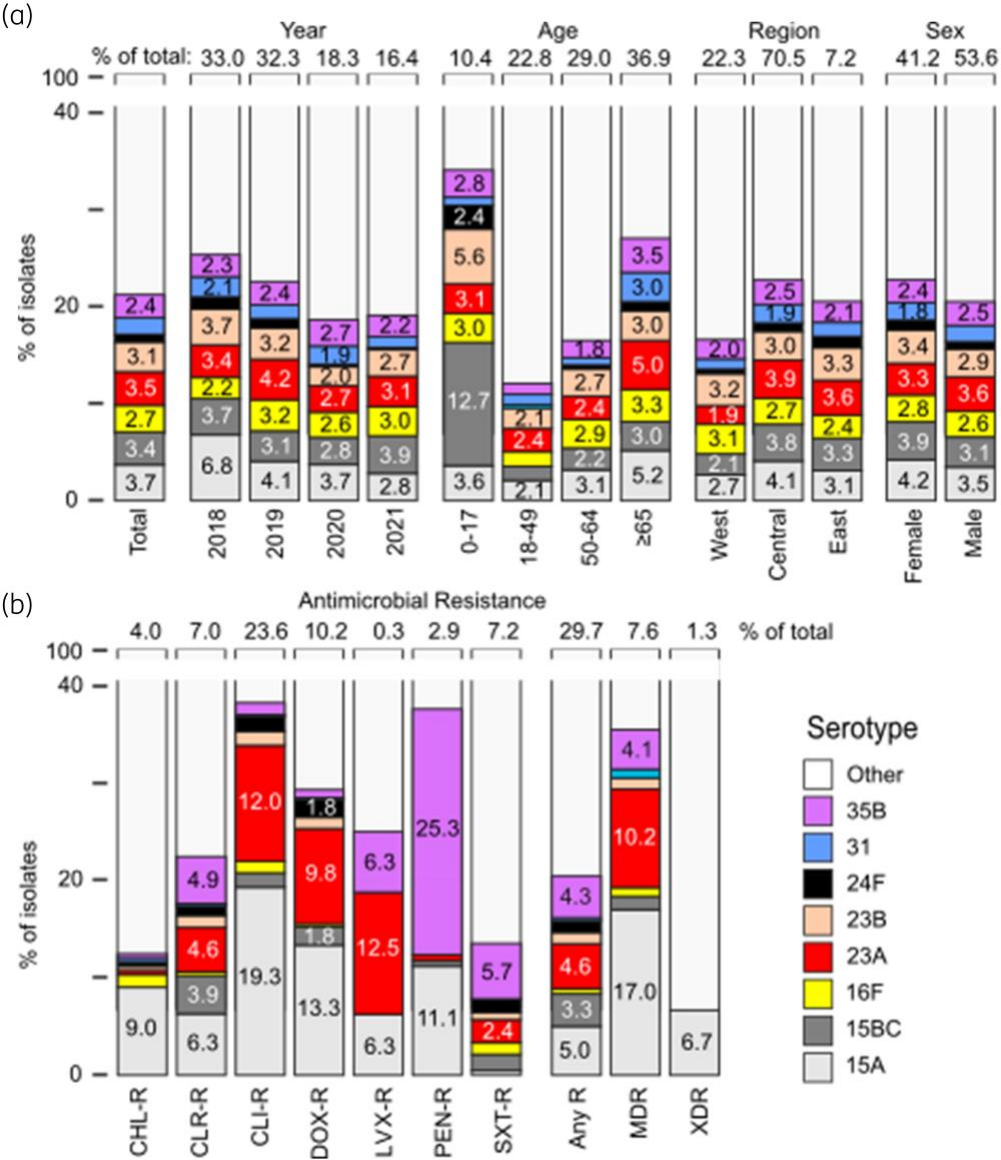
Coverage of IPD isolates by V116 unique serotypes stratified by (a) collection year, patient age group, Canadian geographic region and biological sex, and by (b) antimicrobial resistance phenotype. CHL-R, chloramphenicol resistant; CLR-R, clarithromycin resistant; CLI-R, clindamycin resistant; DOX-R, doxycycline resistant; LVX-R, levofloxacin resistant; PEN-R, penicillin resistant; SXT-R, trimethoprim/sulfamethoxazole resistant; any R, any resistance.

Table 3 shows the proportions of the eight unique serotypes in V116 (15A, 15C, 16F, 23A, 23B, 24F, 31 and 35B), as well as those shared with PPSV23 by collection year, patient age group, Canadian geographic region and biological sex. The eight unique serotypes in V116 accounted for 21.2% of serotypes for all isolates, and 16.5% and 27.1% of all serotypes isolated from patients aged 50–64 and ≥65 years, respectively (Figure 2). The eight unique serotypes in V116 were found in all Canadian geographic areas (range, 16.6%–22.8%) and in both biological sexes (20.5% in males and 22.8% in females). The shared serotypes of V116 and PPSV23 accounted for 11.9% of all isolates, 15.3% in patients aged 50–64 years, and 10.4% in patients aged ≥65 years. The shared serotypes of V116 and PPSV23 were present in all Canadian regions and in both biological sexes. PCV-20 unique serotypes not contained in V116 accounted for 12.7% of all isolates, 13.6% of isolates from patients aged 50–64 years, and 7.9% of isolates from patients aged ≥65 years (Table S1). V116 coverage by Canadian geographic region ranged from 74.5% to 83.8% for patients aged 50–64 years and from 77.7% to 80.9% for patients aged ≥65 years (Figure S2).

**Table 3.** Proportion of invasive *S. pneumoniae* isolates with unique V116 serotypes compared with isolates with V116 and PPSV-23 shared serotypes, by collection year, patient age, Canadian geographic region and biological sex

Category (*n*)	V116 unique serotypes, *n* (%)									V116 and PPSV23 shared serotypes *n* (%)			
	15A	15C	16F	23A	23B	24F	31	35B	Total	9N	17F	20	Total
All isolates (5854)	217 (3.7)	198 (3.4)	160 (2.7)	203 (3.5)	179 (3.1)	47 (0.8)	99 (1.7)	140 (2.4)	1243 (21.2)	388 (6.6)	71 (1.2)	235 (4.0)	694 (11.9)
Year													
2018 (1930)	131 (6.8)	72 (3.7)	42 (2.2)	65 (3.4)	71 (3.7)	24 (1.2)	41 (2.1)	44 (2.3)	490 (25.4)	109 (5.6)	29 (1.5)	51 (2.6)	189 (9.8)
2019 (1893)	77 (4.1)	59 (3.1)	61 (3.2)	79 (4.2)	61 (3.2)	18 (1.0)	27 (1.4)	46 (2.4)	428 (22.6)	135 (7.1)	21 (1.1)	57 (3.0)	213 (11.3)
2020 (1073)	40 (3.7)	30 (2.8)	28 (2.6)	29 (2.7)	21 (2.0)	3 (0.3)	20 (1.9)	29 (2.7)	200 (18.6)	75 (7.0)	10 (0.9)	46 (4.3)	131 (12.2)
2021 (958)	27 (2.8)	37 (3.9)	29 (3.0)	30 (3.1)	26 (2.7)	2 (0.2)	11 (1.1)	21 (2.2)	183 (19.1)	69 (7.2)	11 (1.1)	81 (8.5)	161 (16.8)
Age group, years													
0 to <18 (607)	22 (3.6)	77 (12.7)	18 (3.0)	19 (3.1)	34 (5.6)	15 (2.5)	5 (0.8)	17 (2.8)	207 (34.1)	10 (1.6)	3 (0.5)	9 (1.5)	22 (3.6)
18–49 (1332)	28 (2.1)	19 (1.4)	20 (1.5)	32 (2.4)	28 (2.1)	5 (0.4)	14 (1.1)	15 (1.1)	161 (12.1)	95 (7.1)	14 (1.1)	73 (5.5)	182 (13.7)
50–64 (1696)	54 (3.2)	38 (2.2)	50 (2.9)	41 (2.4)	47 (2.8)	7 (0.4)	13 (0.8)	30 (1.8)	280 (16.5)	144 (8.5)	15 (0.9)	101 (6.0)	260 (15.3)
≥65 (2161)	112 (5.2)	64 (3.0)	72 (3.3)	109 (5.0)	66 (3.1)	20 (0.9)	66 (3.1)	76 (3.5)	585 (27.1)	136 (6.3)	38 (1.8)	51 (2.4)	225 (10.4)
Region^a^													
Western (1307)	35 (2.7)	28 (2.1)	40 (3.1)	25 (1.9)	43 (3.3)	6 (0.5)	14 (1.1)	26 (2.0)	217 (16.6)	77 (5.9)	15 (1.1)	86 (6.6)	178 (13.6)
Central (4128)	169 (4.1)	156 (3.8)	110 (2.7)	163 (3.9)	122 (3.0)	36 (0.9)	79 (1.9)	105 (2.5)	940 (22.8)	263 (6.4)	52 (1.3)	128 (3.1)	443 (10.7)
Eastern (419)	13 (3.1)	14 (3.3)	10 (2.4)	15 (3.6)	14 (3.3)	5 (1.2)	6 (1.4)	9 (2.1)	86 (20.5)	48 (11.5)	4 (1.0)	21 (5.0)	73 (17.4)
Biological sex													
Female (2415)	102 (4.2)	94 (3.9)	67 (2.8)	79 (3.3)	83 (3.4)	24 (1.0)	44 (1.8)	58 (2.4)	551 (22.8)	138 (5.7)	23 (1.0)	95 (3.9)	256 (10.6)
Male (3140)	109 (3.5)	97 (3.1)	83 (2.6)	112 (3.6)	90 (2.9)	23 (0.7)	53 (1.7)	77 (2.5)	644 (20.5)	225 (7.2)	45 (1.4)	136 (4.3)	406 (12.9)

^a^Western Canada includes Saskatchewan and Manitoba; Central Canada includes Ontario and Quebec; Eastern Canada includes Newfoundland and Labrador, Nova Scotia, Prince Edward Island and New Brunswick.

### Antimicrobial resistance in the eight unique serotypes in V116

Figure 2(a and b) shows the breakdown of the V116 eight unique serotypes by study year, age, geographic location and biological sex, as well as antimicrobial resistance phenotype. Of the eight unique V116 serotypes, 15A demonstrated the highest resistance to chloramphenicol, clarithromycin, clindamycin, doxycycline, resistance to any one single agent, and the highest MDR rate (17.0%) and XDR rate (6.7%). Serotype 23A also demonstrated frequent antimicrobial resistance, as well as the highest rate of resistance to levofloxacin (12.5%) and an MDR rate of 10.2%. Table S2 shows additional antimicrobial resistance data for V116 and PPSV23 shared serotypes. Of the V116 and PPSV23 shared serotypes, antimicrobial resistance ranged from a low of 2.4% for penicillin to a high of 12.5% for levofloxacin. Table S3 shows antimicrobial resistance data for V116 and PCV20 shared serotypes. For these serotypes, resistance ranged from a low of 18.8% for levofloxacin to a high of 61.8% for chloramphenicol, and demonstrated an MDR rate of 34.2% and an XDR rate of 65.3%. Among the isolates with PCV20 unique serotypes, resistance ranged from a low of 7.8% for clindamycin and clarithromycin to a high of 22.4% for penicillin, with an MDR rate of 19.9% and an XDR rate of 26.7%.

## Discussion

Before the COVID-19 pandemic, approximately 100 000 non-invasive pneumococcal pneumonia hospitalizations and 30 000 IPD cases occurred annually among adults in the USA.^22^ This significant disease burden, as well as the evolution of non-vaccine serotypes including antimicrobial non-susceptible serotypes, necessitates the continued development of higher-valency PCVs.^18,21,30–32^ V116 (PCV21) is a novel 21-valent PCV developed specifically for use in adults, covering serotypes first included in PCV13 (3, 6A, 7F, 19A), PCV15 (22F, 33F), PCV20 (8, 10A, 11A, 12F) and PPSV23 (9N, 17F, 20), as well as eight unique serotypes (15A, 15C, 16F, 23A, 23B, 24F, 31, 35B).^14–17,19–21^ V116 contains 4 μg of each pneumococcal polysaccharide individually conjugated to the non-toxic diphtheria toxoid CRM197 carrier protein.^19^

Our study showed that V116 demonstrated greater coverage of IPD isolates than PCV20 and PPSV23 for patients aged 50–64 and ≥65 years (Table 1). In patients aged ≥65 years, V116 offered the highest coverage at 81.5% (versus 52.2% for PCV20 and 60.0% for PPSV23) (Table 1). Our finding (81.5% vaccine coverage) compares favourably with data from US investigators who reported that serotypes contained in PCV21 caused approximately 80% of IPD cases among adults with indications for vaccination.^21,22^

Earlier studies in adults have compared the immunogenicity of V116 with comparator vaccines (PCV15, PCV20, PPSV23) by measuring opsonophagocytic activity geometric mean titres and percent seroresponders.^16,17,19,20^ In brief, among immunocompetent, pneumococcal vaccine-naive adults aged ≥50 years, V116 met non-inferiority criteria for serotypes shared with comparator vaccines (PPSV23, PCV20).^16,17,19,20^ V116 elicited significantly higher immune responses for all V116 unique serotypes except 15C, which is likely the result of the presence of serotype 15B in other vaccines generating cross-reactive functional antibodies.^16,17,19,20^ It bears repeating, however, that no data exist on whether the immunological cross-reactive antibodies of serotypes 15B and 15C result in clinical cross-protection. Among immunocompetent adults aged ≥50 years who had previously received a pneumococcal vaccine (PCV13 or PPSV23) ≥1 year before enrolment, V116 demonstrated comparable immunogenicity for shared serotypes and was immunogenic for unique serotypes compared with PPSV23 or PCV15. Among adults who had previously received PPSV23 followed by or preceded by PCV13, PPSV23 preceded by PCV15, or PCV15 alone, V116 was immunogenic for all serotypes.^19^ V116 demonstrated comparable safety to comparator vaccines.^16,17,19,20^ Available V116 clinical data are solely immunological and no clinical efficacy of clinical effectiveness data are available.^33,34^ Thus, whether V116 immunogenicity will in fact confer clinical effectiveness needs to be proven in real-world studies.

In the current study we observed that V116 coverage of antimicrobial-resistant, MDR and XDR serotypes was significantly greater compared with PCV20 and PPSV23, including the MDR and XDR serotypes 19A, 15A, 23A and 35B, which are commonly identified as MDR serotypes (Table 2).^23,25,32^ It should be stated that other definitions of MDR and XDR *S. pneumoniae* exist and thus our vaccine coverage data of MDR and XDR strains should be interpreted with caution.^23^ We also observed that the eight unique serotypes in V116 represented 27.1% of all serotypes in patients aged ≥65 years (Table 3), which compares favourably with data from the USA that showed 20%–30% of IPD was due to the eight new serotypes contained in PCV21.^22^

ACIP recommends that adults aged ≥50 years, as well as adults aged 19–49 years with certain risk conditions for pneumococcal disease who have not received a PCV or whose vaccination history is unknown, receive either PCV20 or PCV15 followed by PPSV23 or V116, with no preferential recommendation for which vaccine to use.^13,22^ Our data suggest that V116 (76.3% and 81.5%) would provide the broadest vaccine coverage in adults aged 50–64 and ≥65 years, respectively, followed by PPSV23 (75.1% and 60.0%) and PCV20 (60.2% and 52.2%) (Table 1). In adults aged 19–49 years with certain risk conditions for pneumococcal disease, PCV20 and PCV21 (V116) coverage was similar at 69.2% and 72.3%, respectively (Table 1). Our data also suggest that in patients who have already received PCV13, PCV15, PCV20 or PPSV23, V116 may offer an additional 55.5%, 41.7%, 29.3% or 21.5% coverage (Table 1). It should, however, be mentioned that currently no recommendations exist for patients who have received PCV20 that they should also receive PCV21 (V116).^35^ In Canada, NACI has made a preferential recommendation of PCV20 for adults aged ≥65 years; thus, in adults aged ≥65 years who have already received PCV20, V116 may offer 29.3% additional coverage (Table 1).^12^

The SAVE study has several limitations that require mentioning. The lack of representation of western Canada’s two most populated provinces, Alberta and British Columbia, mitigate any firm conclusions being drawn from regional comparisons. An important limitation of the SAVE study (2018–21) is that serotype evolution continues in Canada and other jurisdictions (post the COVID-19 pandemic) and thus vaccine coverage needs to be assessed annually based on serotype distribution regionally and nationally. During the 2020 and 2021 years of the COVID-19 pandemic, a reduced number of isolates were received and tested in the SAVE study, which could have impacted the results. Recent data post COVID-19 from Canada show that serotypes 4 and 9V, not covered by V116, are increasing in Canadian adults thus lowering V116 coverage compared with PCV20.^35^ In fact, serotype 4 has been reported to be re-emerging also in some regions of the USA, and in some populations may represent ≥30% of pneumococcal disease, meaning that in these populations PCV20 alone or PCV15 and PPSV23 in series may provide broader serotype coverage compared with V116.^22,35^ This clinical guidance speaks to the critical need for ongoing pneumococcal surveillance to assess serotype evolution including assessing the proportion of non-PCV21 (V116) serotypes that are contained in PCV20. Some serotypes such as serotype 3 continue to cause disease despite being a component of pneumococcal vaccines.^36^ Although V116 has demonstrated a greater immunological response (1.55 times the geometric mean titre ratio) than PCV20 versus serotype 3, it is unknown if this translates into increased effectiveness.^19^ Lastly, it is unclear how V116 compares with PCV20 in terms of cost.^21,22^ Different models cannot agree whether V116 is cost-saving compared with PCV20.^21,22^

In summary, V116 provided significantly greater coverage of IPD isolates than PCV15, PCV20 and PPSV23 for different age groups, including adults aged 50–64 and ≥65 years, across all Canadian geographic regions, and for all individual antimicrobial resistance phenotypes, as well as MDR isolates. In patients aged ≥65 years, vaccine coverage was 39.8% for PCV15, 52.2% for PCV20, 60.0% for PPSV23, 81.5% for V116, and 89.3% for V116 combined with PCV20. The eight unique serotypes in V116 represented 27.1% of all serotypes in patients aged ≥65 years.

## Supplementary Material

dkaf085_Supplementary_Data

## Supplementary data

Figures S1 and S2 and Tables S1–S3 are available as Supplementary data at *JAC* Online.

## References

[1] Ginsburg AS , Klugman KP. Vaccination to reduce antimicrobial resistance. Lancet Glob Health 2017; 5: e1176–7. 10.1016/S2214-109X(17)30364-9 29128252

[2] WHO . Estimating the Impact of Vaccines in Reducing Antimicrobial Resistance and Antibiotic use: Technical Report. 2024. https://www.who.int/publications/i/item/9789240098787.

[3] Mullins LP , Mason E, Winter K et al Vaccination is an integral strategy to combat antimicrobial resistance. PLoS Pathog 2023; 19: e1011379. 10.1371/journal.ppat.1011379 37319164 PMC10270329

[4] van Heuvel L , Caini S, Duckers MLA et al Assessment of the inclusion of vaccination as an intervention to reduce antimicrobial resistance in AMR national action plans: a global review. Global Health 2022; 18: 85. 10.1186/s12992-022-00878-6 36253789 PMC9574789

[5] Scelfo C , Menzella F, Fontana M et al Pneumonia and invasive pneumococcal diseases: the role of pneumococcal conjugate vaccine in the era of multi-drug resistance. Vaccines (Basel) 2021; 9: 420. 10.3390/vaccines9050420 33922273 PMC8145843

[6] Bettinger JA , Scheifele DW, Kellner JD et al The effect of routine vaccination on invasive pneumococcal infections in Canadian children, Immunization Monitoring Program, Active 2000–2007. Vaccine 2010; 28: 2130–6. 10.1016/j.vaccine.2009.12.026 20044050

[7] Zhanel GG , Lynch JP, Adam HJ. *Streptococcus pneumoniae* serotyping and antimicrobial susceptibility: assessment for vaccine efficacy in Canada after the introduction of PCV13. J Antimicrob Chemother 2023; 78 Suppl 1: 2–7. 10.1093/jac/dkad064 37130585

[8] Schellenberg JJ , Adam HJ, Baxter MR et al Comparison of PCV10, PCV13, PCV15, PCV20 and PPSV23 vaccine coverage of invasive *Streptococcus pneumoniae* isolate serotypes in Canada: the SAVE study, 2011–20. J Antimicrob Chemother 2023; 78 Suppl 1: 37–47. 10.1093/jac/dkad068 37130588

[9] Golden AR , Adam HJ, Zhanel GG et al Invasive *Streptococcus pneumoniae* in Canada, 2011–2014: characterization of new candidate 15-valent pneumococcal conjugate vaccine serotypes 22F and 33F. Vaccine 2016; 34: 2527–30. 10.1016/j.vaccine.2016.03.058 27085174

[10] Hink RK , Adam HJ, Golden AR et al Comparison of PCV-10 and PCV-13 vaccine coverage for invasive pneumococcal isolates obtained across Canadian geographic regions, SAVE 2011 to 2017. Diagn Microbiol Infect Dis 2021; 99: 115282. 10.1016/j.diagmicrobio.2020.115282 33341491

[11] Golden AR , Fear T, Baxter M et al Invasive pneumococcal disease caused by serotypes 22F and 33F in Canada: the SAVE study 2011–2018. Diagn Microbiol Infect Dis 2021; 101: 115447. 10.1016/j.diagmicrobio.2021.115447 34192638

[12] National Advisory Committee on Immunization (NACI) . An Advisory Committee Statement (ACS): Public health level recommendations on the use of pneumococcal vaccines in adults, including the use of 15-valent and 20-valent conjugate vaccines. 2023. https://www.canada.ca/en/public-health/services/immunization/national-advisory-committee-on-immunization-naci/public-health-level-recommendations-use-pneumococcal-vaccines-adults-including-use-15-valent-20-valent-conjugate-vaccines.html.

[13] Advisory Committee on Immunization Practice (ACIP), CDC . Recommended adult immunization schedules, ages 19 and older. 2024. www.cdc.gov/vaccines/vpd/pneumo/hcp/pneumoapp.html.

[14] Masomian M , Ahmad Z, Gew LT et al Development of next generation *Streptococcus pneumoniae* vaccines conferring broad protection. Vaccines (Basel) 2020; 8: 132. 10.3390/vaccines8010132 32192117 PMC7157650

[15] Curry S , Kaufhold RM, Monslow MA et al Preclinical evaluation of an investigational 21-valent pneumococcal conjugate vaccine, V116, in adult-rhesus monkey, rabbit, and mouse models. Vaccine 2023; 41: 903–13. 10.1016/j.vaccine.2022.12.017 36566163

[16] Platt H , Omole T, Cardona J et al Safety, tolerability, and immunogenicity of a 21-valent pneumococcal conjugate vaccine, V116, in healthy adults: phase 1/2, randomised, double-blind, active comparator-controlled, multicentre, US-based trial. Lancet Infect Dis 2023; 23: 233–46. 10.1016/S1473-3099(22)00526-6 36116461

[17] Haranaka M , Yono M, Kishino H et al Safety, tolerability, and immunogenicity of a 21-valent pneumococcal conjugate vaccine, V116, in Japanese healthy adults: a phase I study. Hum Vaccine Immunother 2023; 19: 2228162. 10.1080/21645515.2023.2228162 PMC10316726 37389808

[18] Self WH , Johnson KD, Resser JJ et al Prevalence, clinical severity and serotype distribution of pneumococcal pneumonia among adults hospitalized with community acquired pneumonia in Tennessee and Georgia, 2018–2022. Clin Infect Dis 2024; 79: 838–47. 10.1093/cid/ciae316 39016606 PMC11478805

[19] Scott P , Haranaka M, Choi JH et al A phase 3 clinical study to evaluate safety tolerability and immunogenicity of V116 in pneumococcal vaccine-experienced adults 50 years of age and older (STRIDE-6). Clin Infect Dis 2024; 79: 1366–74. 10.1093/cid/ciae383 39082735 PMC11650886

[20] Platt HL , Bruno C, Buntinx E et al Safety, tolerability, and immunogenicity of an adult pneumococcal conjugate vaccine, V116 (STRIDE-3): a randomised, double-blind, active comparator controlled, international phase 3 trial. Lancet Infect Dis 2024; 24: 1141–50. 10.1016/S1473-3099(24)00344-X. 38964361

[21] Yi Z , Johnson KD, Owusu-Edusei K. Lifetime health and economic burden of invasive pneumococcal diseases attributable to V116 serotypes among adults in the United States. Infect Dis Ther 2024; 13: 1501–14. 10.1007/s40121-024-00988-1 38796565 PMC11220086

[22] Kobayashi M , Leidner AJ, Gierke R et al Use of 21-valent pneumococcal conjugate vaccine among U.S. adults: recommendations of the Advisory Committee on Immunization Practices United States. Morb Mortal Wkly Rep 2024; 73: 793–8. 10.15585/mmwr.mm7336a3 PMC11392227 39264843

[23] Adam HJ , Golden AR, Karlowsky JA et al Analysis of multidrug resistance in the predominant *Streptococcus pneumoniae* serotypes in Canada: the SAVE study, 2011–15. J Antimicrob Chemother 2018; 73: 12–9. 10.1093/jac/dky158 29982572

[24] Alford MA , Karlowsky JA, Adam HJ et al Antimicrobial susceptibility testing of invasive isolates of *Streptococcus pneumoniae* from Canadian patients: the SAVE study, 2011–2020. J Antimicrob Chemother 2023; 78 Suppl 1: 8–16. 10.1093/jac/dkad065 37130584

[25] Adam HJ , Karlowsky JA, Baxter MR et al Analysis of MDR in the predominant *Streptococcus pneumoniae* serotypes in Canada: the SAVE study, 2011–2020. J Antimicrob Chemother 2023; 78 Suppl 1: 17–25. 10.1093/jac/dkad066 37130586

[26] Golden AR , Adam HJ, Karlowsky JA et al Genomic investigation of the most common *Streptococcus pneumoniae* serotypes causing invasive infections in Canada: the SAVE study, 2011–2020. J Antimicrob Chemother 2023; 78 Suppl 1: 26–36. 10.1093/jac/dkad067 37130587

[27] Griffith A , Golden AR, Lefebvre B et al Invasive pneumococcal disease surveillance in Canada, 2021–2022. Can Comm Dis Rpt 2024; 50: 121–34. 10.14745/ccdr.v50i05a02 PMC11147492 38835503

[28] Hao L , Kuttel MM, Ravenscroft N et al *Streptococcus pneumoniae* serotype 15B polysaccharide conjugate elicits a cross-functional immune response against serotype 15C but not 15A. Vaccine 2022; 40: 4872–80. 10.1016/j.vaccine.2022.06.041 35810060

[29] Kaur R , Gonzalez E, Pham M et al Naturally-induced serum antibody levels in children to pneumococcal polysaccharide 15B that correlate with protection from nasopharyngeal colonization but anti-serotype 15B antibody has low functional cross-reactivity with serotype 15C. Vaccine 2023; 41: 7265–73. 10.1016/j.vaccine.2023.10.054 37925318

[30] Ladhani SN , Collins S, Djennad A et al Rapid increase in non-vaccine serotypes causing invasive pneumococcal disease in England and Wales, 2000–17: a prospective national observational cohort study. Lancet Infect Dis 2018; 18: 441–51. 10.1016/S1473-3099(18)30052-5 29395999

[31] Lochen A , Croucher NJ, Anderson RM. Divergent serotype replacement trends and increasing diversity in pneumococcal disease in high income settings reduce the benefit of expanding vaccine valency. Sci Rep 2020; 10: 18977. 10.1038/s41598-020-75691-5 33149149 PMC7643077

[32] Suaya JA , Mendes RE, Sings HL et al *Streptococcus pneumoniae* serotype distribution and antimicrobial nonsusceptibility trends among adults with pneumonia in the United States, 2009–2017. J Infect 2020; 81: 557–66. 10.1016/j.jinf.2020.07.035 32739491

[33] Young Song J , Moseley MA, Burton RL et al Pneumococcal vaccine and opsonic pneumococcal antibody. J Infect Chemother 2013; 19: 412–25. 10.1007/s10156-013-0601-1 23657429 PMC3692352

[34] Romero-Steiner S , Frasch CE, Carlone G et al Use of opsonophagocytosis for serological evaluation of pneumococcal vaccines. Clin Vaccine Immunol 2006; 13: 165–9. 10.1128/CVI.13.2.165-169.2006 16467321 PMC1391943

[35] National Advisory Committee on Immunization (NACI) . An Advisory Committee Statement (ACS): Recommendations on the use of pneumococcal vaccines in adults, including PNEU-C-21. 2024. https://www.canada.ca/en/public-health/services/publications/vaccines-immunization/national-advisory-committee-immunization-summary-recommendations-use-pneumococcal-vaccines-adults-pneu-c-21.html. 10.14745/ccdr.v51i101112a01 PMC12795405 41531856

[36] Schellenberg JJ , Adam HJ, Baxter MR et al Phenotypic and molecular characterization of *Streptococcus pneumoniae* serotype 3 isolates from blood and respiratory samples in Canada: CANWARD 2007–21. J Antimicrob Chemother 2024; 79: 2653–61. 10.1093/jac/dkae272 39092981 PMC11442004

