# Supplementary material for: Comparing serotype coverage of pneumococcal vaccines with PCV21 (V116), a new 21-valent conjugate pneumococcal vaccine, and the epidemiology of its eight unique Streptococcus pneumoniae serotypes (15A, 15C, 16F, 23A, 23B, 24F, 31 and 35B) causing invasive pneumococcal disease in adult patients in Canada: SAVE study, 2018–21
Source: J Antimicrob Chemother. 2025 Mar 25;80(5):1377–85. doi: 10.1093/jac/dkaf085 (PMC12046396; doi:10.1093/jac/dkaf085)
Supplement: dkaf085_Supplementary_Data [file dkaf085_supplementary_data.docx]

**SUPPLEMENTARY DATA**

**Supplementary Table S1**. Proportion of invasive *S. pneumoniae* isolates with serotypes shared by V116 and PCV-20, PCV20 unique serotypes, and non-vaccine serotypes stratified by collection year, patient age, Canadian geographic region, and biological sex.

| **Category (n)** | **V116 and PCV20 shared serotypes (%)** | | | | | | | | | | | | | | | | | | | | **PCV20 unique (%)** | **NVS (%)** |
| --- | --- | --- | --- | --- | --- | --- | --- | --- | --- | --- | --- | --- | --- | --- | --- | --- | --- | --- | --- | --- | --- | --- |
|  | **3** | **6A** | | **7F** | | **8** | | **10A** | | **11A** | | **12F** | | **19A** | | **22F** | | **33F** | **Total** | |  |  |
| All isolates (5854) | 645 (11.0) | 19 (0.3) | | 572 (1.4) | | 366 (6.3) | | 104 (1.8) | | 205 (3.5) | | 283 (4.8) | | 230 (3.9) | | 470 (8.0) | | 198 (3.4) | 2603 (44.5) | | 742 (12.7) | 572 (9.8) |
| **Year** |  |  | |  | |  | |  | |  | |  | |  | |  | |  |  | |  |  |
| 2018 (1930) | 237 (12.3) | 11 (0.6) | | 20 (1.0) | | 111 (5.8) | | 39 (2.0) | | 78 (4.0) | | 89 (4.6) | | 98 (5.1) | | 177 (9.2) | | 67 (3.5) | 927 (48.0) | | 176 (9.1) | 206 (10.7) |
| 2019 (1893) | 214 (11.3) | 4 (0.2) | | 24 (1.3) | | 111 (5.9) | | 32 (1.7) | | 64 (3.4) | | 83 (4.4) | | 67 (3.5) | | 180 (9.5) | | 86 (4.5) | 865 (45.7) | | 205 (10.8) | 182 (9.6) |
| 2020 (1073) | 115 (10.7) | 2 (0.2) | | 24 (2.2) | | 87 (8.1) | | 23 (2.1) | | 33 (3.1) | | 55 (5.1) | | 37 (3.4) | | 66 (6.2) | | 33 (3.1) | 475 (44.3) | | 182 (17.0) | 85 (7.9) |
| 2021 (958) | 79 (8.2) | 2 (0.2) | | 15 (1.6) | | 57 (5.9) | | 10 (1.0) | | 30 (3.1) | | 56 (5.8) | | 28 (2.9) | | 47 (4.9) | | 12 (1.3) | 336 (35.1) | | 179 (18.7) | 99 (10.3) |
| **Age Group** |  |  | |  | |  | |  | |  | |  | |  | |  | |  |  | |  |  |
| 0-<18 (607) | 56 (9.2) | 2 (0.3) | | 5 (0.8) | | 16 (2.6) | | 19 (3.1) | | 17 (2.8) | | 11 (1.8) | | 36 (5.9) | | 64 (10.5) | | 30 (4.9) | 256 (42.2) | | 41 (6.8) | 81 (13.3) |
| 18-49 (1332) | 131 (9.8) | 1 (0.1) | | 40 (3.0) | | 137 (10.3) | | 15 (1.1) | | 27 (2.0) | | 125 (9.4) | | 42 (3.2) | | 72 (5.4) | | 35 (2.6) | 625 (46.9) | | 292 (21.9) | 72 (5.4) |
| 50-64 (1696) | 208 (12.3) | 8 (0.5) | | 21 (1.2) | | 124 (7.3) | | 25 (1.5) | | 66 (3.9) | | 93 (5.5) | | 65 (3.8) | | 116 (6.8) | | 46 (2.7) | 772 (45.5) | | 231 (13.6) | 153 (9.0) |
| ≥65 (2161) | 243 (11.2) | 8 (0.4) | | 16 (0.7) | | 85 (3.9) | | 44 (2.0) | | 92 (4.3) | | 49 (2.3) | | 84 (3.9) | | 216 (10.0) | | 84 (3.9) | 921 (42.6) | | 170 (7.9) | 260 (12.0) |
| **Region^a^** |  |  | |  | |  | |  | |  | |  | |  | |  | |  |  | |  |  |
| Western (1307) | 102 (7.8) | 0 | | 13 (1.0) | | 122 (9.3) | | 14 (1.1) | | 31 (2.4) | | 120 (9.2) | | 31 (2.4) | | 108 (8.3) | | 32 (2.4) | 573 (43.8) | | 257 (19.7) | 82 (6.3) |
| Central (4128) | 507 (12.3) | 18 (0.4) | | 62 (1.5) | | 215 (5.2) | | 82 (2.0) | | 150 (3.6) | | 162 (3.9) | | 179 (4.3) | | 315 (7.6) | | 150 (3.6) | 1840 (44.6) | | 459 (11.1) | 446 (10.8) |
| Eastern (419) | 36 (8.6) | 1 (0.2) | | 8 (1.9) | | 29 (6.9) | | 8 (1.9) | | 24 (5.7) | | 1 (0.2) | | 20 (4.8) | | 47 (11.2) | | 16 (3.8) | 190 (45.3) | | 26 (6.2) | 44 (10.5) |
| **Biological sex** |  | |  | |  | |  | |  | |  | |  | |  | |  | | |  |  |  |
| Female (2415) | 280 (11.6) | 5 (0.2) | | 30 (1.2) | | 138 (5.7) | | 41 (1.7) | | 101 (4.2) | | 98 (4.1) | | 100 (4.1) | | 202 (8.4) | | 104 (4.3) | 1099 (45.5) | | 268 (11.1) | 241 (10.0) |
| Male (3140) | 319 (10.2) | 14 (0.4) | | 48 (1.5) | | 218 (6.9) | | 57 (1.8) | | 90 (2.9) | | 166 (5.3) | | 118 (3.8) | | 240 (7.6) | | 81 (2.6) | 1351 (43.0) | | 427 (13.6) | 312 (9.9) |

^a^Western Canada includes Saskatchewan and Manitoba; Central Canada includes Ontario and Quebec; Eastern Canada includes Newfoundland and Labrador, Nova Scotia, Prince Edward Island and New Brunswick.

Abbreviations: NVS, non-vaccine serotype.

**Supplementary Table S2**. Proportion of invasive *S. pneumoniae* isolates with V116 unique and V116 and PPSV-23 shared serotypes, stratified by antimicrobial resistance phenotype.

| **Category (n)** | **V116 unique serotypes (%)** | | | | | | | | | **V116 and PPSV23 shared serotypes (%)** | | | |
| --- | --- | --- | --- | --- | --- | --- | --- | --- | --- | --- | --- | --- | --- |
|  | **15A** | **15BC** | **16F** | **23A** | **23B** | **24F** | **31** | **35B** | **Total** | **9N** | **17F** | **20** | **Total** |
| CHL-R (233) | 21 (9.0) | 0 | 3 (1.3) | 1 (0.4) | 1 (0.4) | 1 (0.4) | 1 (0.4) | 1 (0.4) | 29 (12.4) | 2 (0.9) | 0 | 4 (1.7) | 6 (2.6) |
| CLI-R (410) | 79 (19.3) | 6 (1.5) | 5 (1.2) | 49 (12.0) | 6 (1.5) | 6 (1.5) | 1 (0.2) | 5 (1.2) | 157 (38.3) | 7 (1.7) | 4 (1.0) | 4 (1.0) | 15 (3.7) |
| CLA-R (1381) | 87 (6.3) | 54 (3.9) | 5 (0.4) | 63 (4.6) | 17 (1.2) | 13 (0.9) | 4 (0.3) | 67 (4.9) | 310 (22.4) | 33 (2.4) | 5 (0.4) | 7 (0.5) | 45 (3.3) |
| DOX-R (600) | 80 (13.3) | 11 (1.8) | 2 (0.3) | 59 (9.8) | 7 (1.2) | 11 (1.8) | 1 (0.2) | 5 (0.8) | 176 (29.3) | 16 (2.7) | 7 (1.2) | 6 (1.0) | 29 (4.8) |
| LEV-R (16) | 1 (6.3) | 0 | 0 | 2 (12.5) | 0 | 0 | 0 | 1 (6.3) | 4 (25.0) | 2 (12.5) | 0 | 0 | 2 (12.5) |
| PEN-R (170) | 19 (11.2) | 1 (0.6) | 0 | 1 (0.6) | 0 | 0 | 0 | 43 (25.3) | 64 (37.6) | 3 (1.8) | 0 | 1 (0.6) | 4 (2.4) |
| SXT-R (422) | 2 (0.5) | 7 (1.7) | 5 (1.2) | 10 (2.4) | 3 (0.7) | 6 (1.4) | 0 | 24 (5.7) | 57 (13.5) | 8 (1.9) | 0 | 3 (0.7) | 11 (2.6) |
| Any R (1736) | 87 (5.0) | 58 (3.3) | 9 (0.5) | 80 (4.6) | 20 (1.2) | 20 (1.2) | 6 (0.3) | 74 (4.3) | 354 (20.4) | 43 (2.5) | 10 (0.6) | 12 (0.7) | 65 (3.7) |
| MDR (442) | 75 (17.0) | 6 (1.4) | 4 (0.9) | 45 (10.2) | 5 (1.1) | 4 (0.9) | 0 | 18 (4.1) | 157 (35.5) | 10 (2.3) | 3 (0.7) | 4 (0.9) | 17 (3.8) |
| XDR (75) | 5 (6.7) | 0 | 0 | 0 | 0 | 0 | 0 | 0 | 5 (6.7) | 0 | 0 | 0 | 0 |

Abbreviations: CHL-R, chloramphenicol-resistant, CLI-R, clindamycin-resistant, CLA-R, clarithromycin-resistant, DOX-R, doxycycline-resistant, LEV-R, levofloxacin-resistant, PEN-R, penicillin-resistant, SXT-R, trimethoprim/sulfamethoxazole-resistant; Any R, any resistance; MDR, defined as resistance to three or more antimicrobial classes; XDR, defined as resistance to five or more antimicrobial classes.

**Supplementary Table S3**. Proportion of invasive *S. pneumoniae* isolates with serotypes shared by V116 and PCV-20, PCV20 unique serotypes, and non-vaccine serotypes stratified by antimicrobial resistance phenotype.

| **Category (n)** | **V116 and PCV20 shared serotypes (%)** | | | | | | | | | | | **PCV20 unique (%)** | **NVS (%)** |
| --- | --- | --- | --- | --- | --- | --- | --- | --- | --- | --- | --- | --- | --- |
|  | **3** | **6A** | **7F** | **8** | **10A** | **11A** | **12F** | **19A** | **22F** | **33F** | **Total** |  |  |
| CHL-R (233) | 74 (31.8) | 3 (1.3) | 0 | 1 (0.4) | 0 | 5 (2.1) | 38 (16.3) | 4 (1.7) | 16 (6.9) | 3 (1.3) | 144 (61.8) | 44 (18.9) | 10 (4.3) |
| CLI-R (410) | 31 (7.6) | 2 (0.5) | 0 | 0 | 0 | 12 (2.9) | 1 (0.2) | 73 (17.8) | 14 (3.4) | 10 (12.4) | 143 (60.8) | 71 (7.8) | 24 (5.9) |
| CLA-R (1381) | 43 (3.1) | 13 (0.9) | 1 (0.1) | 2 (0.1) | 13 (0.9) | 72 (5.2) | 133 (9.6) | 161 (11.7) | 230 (16.7) | 171 (12.4) | 839 (60.8) | 108 (7.8) | 79 (5.7) |
| DOX-R (600) | 82 (13.7) | 5 (0.8) | 0 | 3 (0.5) | 1 (0.2) | 10 (1.7) | 73 (12.2) | 68 (11.3) | 9 (1.5) | 7 (1.2) | 258 (43.0) | 93 (15.5) | 44 (7.3) |
| LEV-R (16) | 0 | 0 | 0 | 0 | 0 | 1 (6.3) | 0 | 1 (6.3) | 1 (6.3) | 0 | 3 (18.8) | 3 (18.8) | 4 (25.0) |
| PEN-R (170) | 0 | 2 (1.2) | 1 (0.6) | 0 | 0 | 2 (1.2) | 1 (0.6) | 46 (27.1) | 2 (1.2) | 0 | 54 (31.8) | 38 (22.4) | 10 (5.9) |
| SXT-R (422) | 1 (0.2) | 1 (0.2) | 1 (0.2) | 1 (0.2) | 2 (0.5) | 30 (7.1) | 68 (16.1) | 57 (13.5) | 5 (1.2) | 37 (8.8) | 203 (48.1) | 48 (11.4) | 103 (24.4) |
| Any R (1736) | 94 (5.4) | 14 (0.8) | 3 (0.2) | 6 (0.3) | 15 (0.9) | 90 (5.2) | 205 (11.8) | 166 (9.6) | 236 (13.6) | 175 (10.1) | 1004 (57.8) | 134 (7.7) | 179 (10.3) |
| MDR (442) | 31 (7.0) | 3 (0.7) | 0 | 0 | 0 | 10 (2.3) | 25 (5.7) | 64 (14.5) | 10 (2.3) | 8 (1.8) | 151 (34.2) | 88 (19.9) | 29 (6.6) |
| XDR (75) | 0 | 1 (1.3) | 0 | 0 | 0 | 6 (8.0) | 1 (1.3) | 39 (52.0) | 2 (2.7) | 0 | 49 (65.3) | 20 (26.7) | 1 (1.3) |

Abbreviations: NVS, non-vaccine serotype; CHL-R, chloramphenicol-resistant, CLI-R, clindamycin-resistant, CLA-R, clarithromycin-resistant, DOX-R, doxycycline-resistant, LEV-R, levofloxacin-resistant, PEN-R, penicillin-resistant, SXT-R, trimethoprim/sulfamethoxazole-resistant; Any R, any resistance; MDR, defined as resistance to three or more antimicrobial classes; XDR, defined as resistance to five or more antimicrobial classes.

**
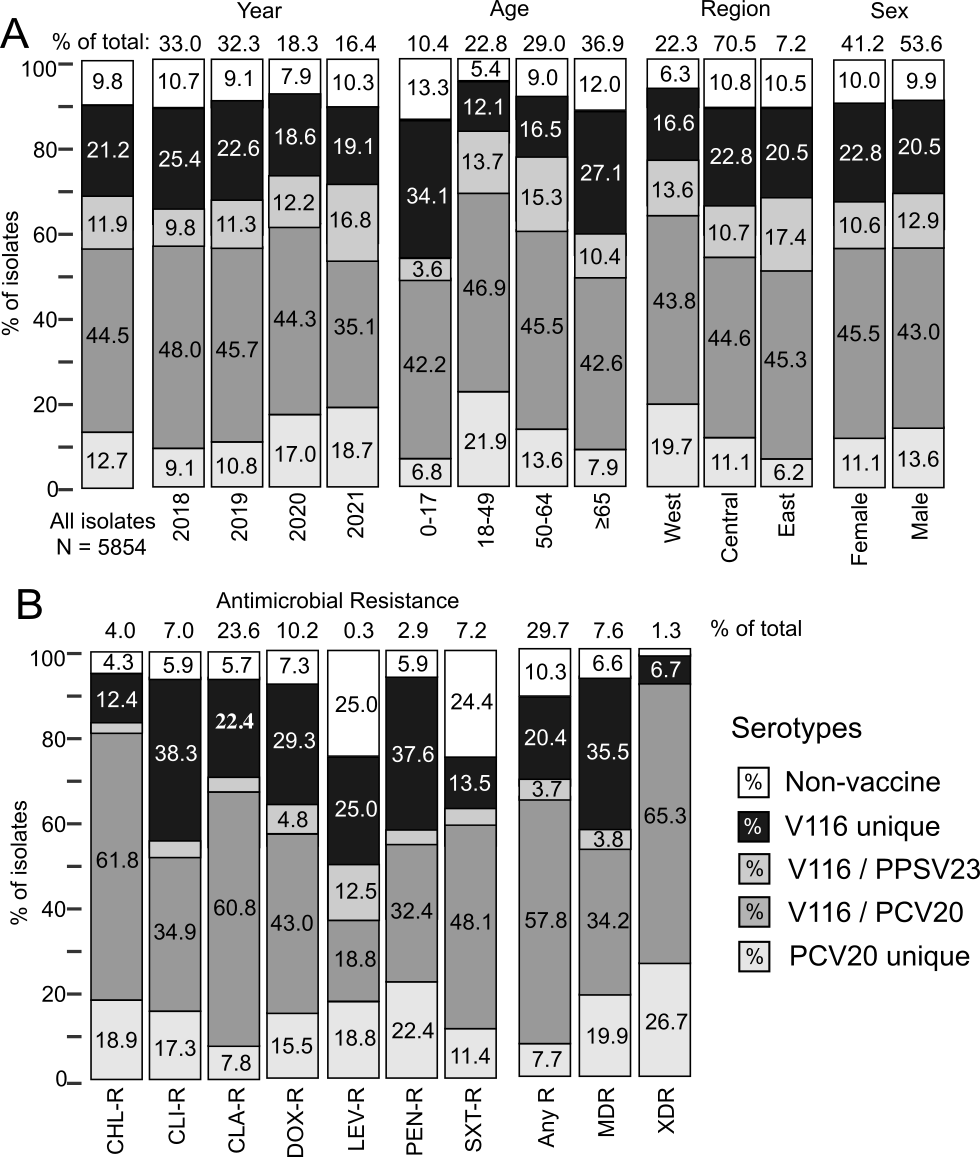
**

**Supplementary Figure S1.** Coverage of IPD isolates by serotypes unique to V116, shared by V116 and PPSV23, and shared by V116 and PCV20 stratified by A) collection year, patient age group, Canadian geographic region, and biological sex and by B) antimicrobial resistance phenotype. Non-vaccine and PCV20 unique isolates are also shown.

Abbreviations: CHL-R, chloramphenicol-resistant, CLA-R, clarithromycin-resistant, CLI-R, clindamycin-resistant, DOX-R, doxycycline-resistant, LEV-R, levofloxacin-resistant, PEN-R, penicillin-resistant, SXT-R, trimethoprim/sulfamethoxazole-resistant; Any R, any resistance; MDR, defined as resistance to three or more antimicrobial classes; XDR, defined as resistance to five or more antimicrobial classes.


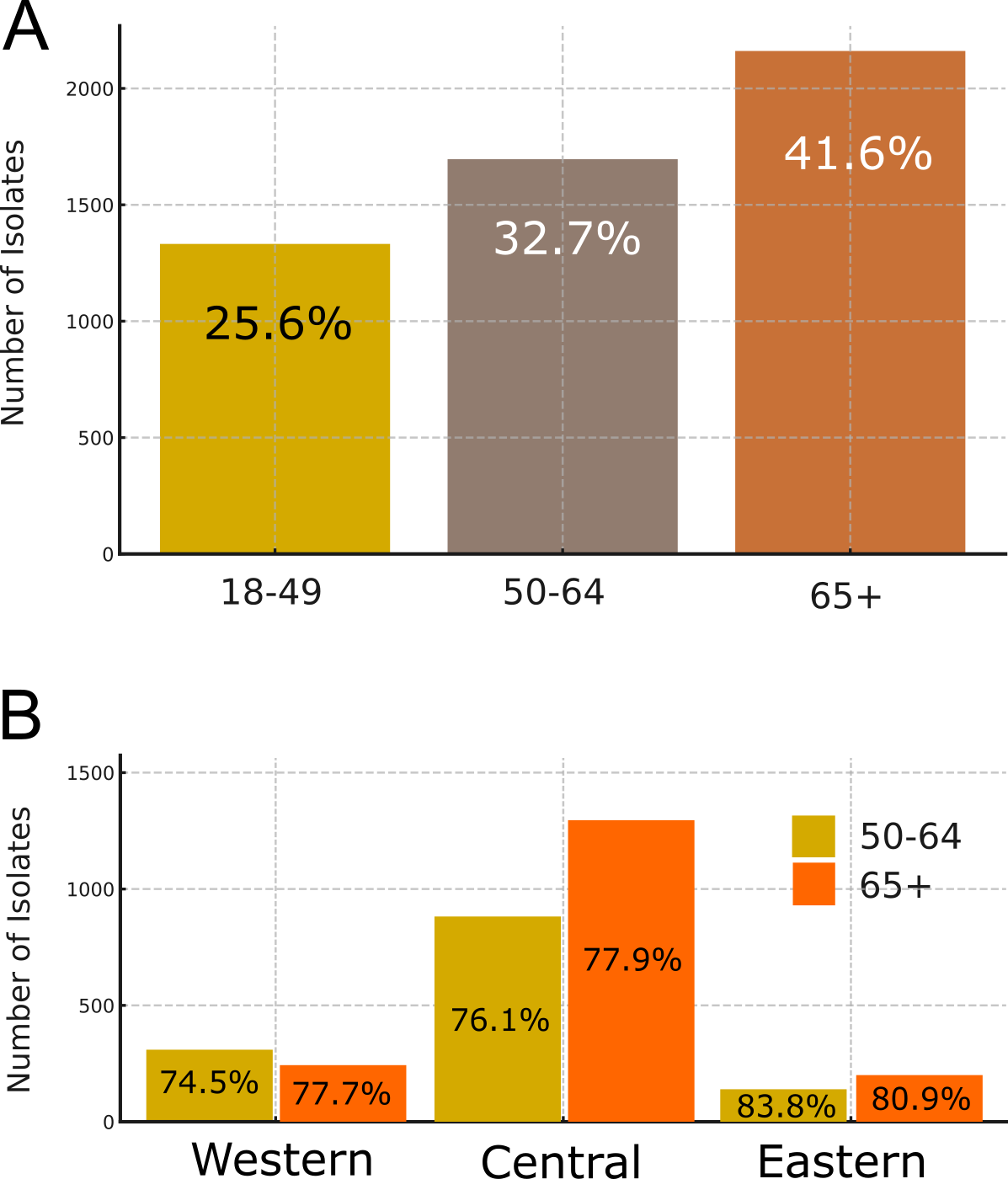


**Supplementary Figure S2.** A) Percentage of all IPD isolates from adults (≥18 years) from 2018 to 2021 stratified by three age groups and B) Percentage of IPD isolates from adults aged 50-64 and ≥65 years covered by V116 across aged 50-64 and ≥65 years the three Canadian geographic regions. Percent values in B) indicate percent serotype coverage by V116 of total isolates for individual age groups in each region.
